# Dynamic Changes in the Expression of Interferon-Stimulated Genes in Joints of SPF Chickens Infected With Avian Reovirus

**DOI:** 10.3389/fvets.2021.618124

**Published:** 2021-02-05

**Authors:** Sheng Wang, Liji Xie, Zhixun Xie, Lijun Wan, Jiaoling Huang, Xianwen Deng, Zhi qin Xie, Sisi Luo, Tingting Zeng, Yanfang Zhang, Minxiu Zhang, Lei Zhou

**Affiliations:** ^1^State Key Laboratory for Conservation and Utilization of Subtropical Agro-Bioresources, College of Animal Science and Technology, Guangxi University, Nanning, China; ^2^Guangxi Key Laboratory of Veterinary Biotechnology, Guangxi Veterinary Research Institute, Nanning, China

**Keywords:** avian reovirus, joints, interferon-stimulated genes, expression, chickens

## Abstract

Avian reovirus (ARV) can induce many diseases as well as immunosuppression in chickens, severely endangering the poultry industry. Interferons (IFNs) play an antiviral role by inducing the expression of interferon-stimulated genes (ISGs). The effect of ARV infection on the expression of host ISGs is unclear. Specific-pathogen-free (SPF) chickens were infected with ARV strain S1133 in this study, and real time quantitative PCR was used to detect changes in the dynamic expression of IFNs and common ISGs in joints of SPF chickens. The results showed that the transcription levels of *IFN*A, *IFNB*, and several ISGs, including myxovirus resistance (*MX*), interferon-induced transmembrane protein 3 (*IFITM3*), protein kinase R (*PKR*), oligoadenylate synthase (*OAS*), interferon-induced protein with tetratricopeptide repeats 5 (*IFIT5*), interferon-stimulated gene 12 (*ISG12*), virus inhibitory protein (*VIPERIN*), interferon-alpha-inducible protein 6 (*IFI6*), and integrin-associated protein (*CD47*), were upregulated in joints on days 1–7 of infection (the levels of increase of *MX, IFIT5, OAS, VIPERIN, ISG12*, and *IFI6* were the most significant, at hundreds-fold). In addition, the expression levels of the ISGs encoding zinc finger protein 313 (*ZFP313*), and DNA damage–inducible transcript 4 (*DDIT4*) increased suddenly on the 1st or 2nd day, then decreased to control levels. The ARV viral load in chicken joints rapidly increased after 1 day of viral challenge, and the viral load remained high within 6 days of viral challenge. The ARV viral load sharply decreased starting on day 7. These results indicate that in SPF chicken joints, many ISGs have mRNA expression patterns that are basically consistent with the viral load in joints. IFNA, IFNB, and the ISGs MX, IFITM3, PKR, OAS, IFIT5, ISG12, VIPERIN, IFI6, and CD47 play important roles in defending against ARV invasion, inhibiting ARV replication and proliferation, and promoting virus clearance. These results enrich our understanding of the innate immune response mechanisms of hosts against ARV infection and provide a theoretical basis for prevention and control of ARV infection.

## Introduction

Avian reovirus (ARV) belongs to the genus Orthoreovirus, family Reoviridae. It mainly causes viral arthritis, chronic respiratory diseases, reduction in egg production, and runting-stunting syndrome and causes severe immunosuppression, all of which bring great economic losses to the poultry industry (1–3). Chicken susceptibility to ARV infection is age-dependent, with older birds being more resistant to both infection and viral-induced lesions. Most birds appear to be infected via the fecal-oral route, but also through the respiratory tract and through skin lesions (4). Histological analyses have show that, major gross pathological lesions included marked swelling, edema, and hemorrhages. Serous exudate was present between the tendons and hock joint. Histological examination demonstrated necrosis and inflammation of muscle fibers, and mixed inflammatory infiltrate was observed in subcutaneous tissue and tendon sheaths (5, 6).

After infection with virus, the innate immune signaling pathways of host are first activated. Type I interferons (IFNs) are important cytokines that play important roles in inhibiting viral replication, regulating immune functions, and inhibiting malignant cell carcinogenesis (7, 8). IFNs cannot directly exert antiviral functions, but they initiate the downstream Janus kinase (JAK)–signal transducer and activator of transcription (STAT) signaling pathway by binding to their receptors interferon alpha and beta receptor subunit 1 (IFNAR1) and IFNAR2 to induce the transcription of hundreds of interferon-stimulated genes (ISGs) to carry out antiviral functions (9, 10). IFNs have been applied extensively in treatment of viral diseases. ISGs are the major antiviral executors of IFNs, so further investigation of the effect of IFN-induced ISGs on viral replication is important.

There are many types of ISG proteins with many different biological activities. Different ISGs can target different stages of viral replication to inhibit replication and achieve an antiviral effect (11). Common avian ISGs include myxovirus resistance (MX), interferon-induced transmembrane protein 3 (IFITM3), interferon-induced protein with tetratricopeptide repeats 5 (IFIT5), oligoadenylate synthase (OAS), protein kinase R(PKR), virus inhibitory protein (VIPERIN), interferon-stimulated gene 12 (ISG12), zinc finger protein 313 (ZFP313), interferon-alpha-inducible protein 6 (IFI6), integrin-associated protein (CD47), and DNA damage–inducible transcript 4 (DDIT4). MX has inhibitory effects on various viruses, including influenza virus and vesicular stomatitis virus, and plays a role in the viral invasion stage. Its structure is highly conserved between species, and it has a broad specificity of antiviral activities through amino acids at specific sites (12–14). The IFITM family proteins can affect the invasion and release of various viruses because they can exert antiviral effects by reducing membrane fluidity and stability (15, 16). Different IFITM family proteins exhibit different inhibitory effects on different viruses. Chicken fibroblast cells with *IFITM3* gene knockout are more susceptible to influenza A virus, indicating that IFITM3 can inhibit influenza A virus replication (16). IFIT5 is the only member of the chicken IFIT family. It localizes to mitochondria, interacts with retinoic acid–inducible gene I (RIG-1) and mitochondrial antiviral signaling protein (MAVS), and can enhance the innate immune signaling pathway to enhance the antiviral effect (17). OAS and PKR are the first-discovered enzymes that are activated after interaction with double-stranded RNA of viruses. These two enzymes rely on their own functions to inhibit viral protein synthesis and viral infection. Chicken OAS has excellent antiviral activities and localizes to the cytoplasm (18). PKR can be activated by double-stranded RNA produced after viral invasion and replication in cells to exert antiviral functions through various routes (19). The ARV σA protein can antagonize IFN-induced antiviral functions. σA protein exerts its function by downregulating the activity of PKR. σA protein can irreversibly interact with double-stranded RNA to block PKR activation and effectively prevent ARV from being recognized by the cellular immune system (20, 21). VIPERIN has broad antiviral activities (22). It mainly localizes to the endoplasmic reticulum (ER) and lipid droplets. *In vivo* and *in vitro* findings show that infectious bursa disease virus and influenza virus can induce significant upregulation of chicken *VIPERIN*. ISG12 protein is a newly discovered ISG protein. It mainly localizes to mitochondria and may exert antiviral functions by regulating cell apoptosis and reciprocally regulating type I IFN signaling pathways (23).

The typical symptom of ARV infection is arthritis. There is no report about the effect of ARV infection on ISG gene expression in host joints. The specific ISGs against various virus differ. Investigation of the changes in ISG transcription in host joints after ARV infection would be helpful for identifying the specific anti-ARV ISG. Therefore, this study experimentally infected 7-day-old specific-pathogen-free (SPF) chickens with the ARV S1133 strain to observe the dynamic pathological changes in chicken joints after infection. Real time quantitative polymerase chain reaction (Real time quantitative PCR) was performed to detect the pattern of changes in the viral load in joints after infection and the changes in the expression of IFNs and ISGs at the transcription level. This study aimed to identify the ISG that may affect ARV replication and to provide a theoretical basis for the prevention and control of ARV.

## Materials and Methods

### Ethics Statement

The present study was approved by the Animal Ethics Committee of the Guangxi Veterinary Research Institute. Sample collections were conducted based on the protocol #2019C0406 issued by Animal Ethics Committee of Guangxi Veterinary Research Institution.

### Virus

ARV strain S1133 was purchased from the China Institute of Veterinary Drug Control. Before use, ARV was propagated using SPF chicken embryos via yolk sac inoculation. Viruses were harvested and inoculated into LMH cells. The viral titer was measured by the Reed-Muench method (24).

### Animal Experiments

SPF White Leghorn chicken eggs were purchased from Beijing Boehringer Ingelheim Vital Biotechnology Co., Ltd. (Beijing, China). Eggs were kept in incubators for 21 days until hatching to obtain 72 1-day-old SPF chickens. Hatched 1-day-old SPF chickens were raised in SPF chicken isolators to 7 days of age. Chickens were randomly divided into the experimental group (group A) and the control group (group B), with 36 animals in each group. Each chicken in group A was challenged with 0.1 ml of ARV S1133 containing 10^4^ tissue culture infection doses, 50% endpoint (TCID_50_) through foot pad injection. Each chicken in group B was injected with the same volume of PBS in the foot pad. After virus challenge, the clinical symptoms of the two groups were observed and photographed. At 1, 2, 3, 4, 5, 6, 7, 10, 14, 21, 28, and 35 days postinfection (dpi), three chickens in each group were randomly collected for pathological observation and real time quantitative PCR detection.

### Pathological Observation

Pathological observation was performed on disease lesions of experimental chickens. Joint samples were collected, fixed in 10% formalin solution, and prepared into pathological sections by Guangxi University of Chinese Medicine. Pathological section preparation involved dehydrating the tissue samples, embedding them in paraffin, sectioning, and staining with hematoxylin and eosin to observe histopathological changes of chicken joints.

### RNA Extraction and cDNA Synthesis

Total RNA was extracted from same-weight (30 mg) joint samples (tendon, synovium, and articular cartilage), using the GeneJET RNA Purification Kit (Thermo scientific, USA) per the manufacturer's protocol. The concentration and purity of the total RNA were determined using a NanoDrop ND1000 spectrophotometer (Thermo Scientific, Boston, MA, USA). The extracted RNA of different samples were formulated to the same concentration (150 ng/μL), and was reverse-transcribed into cDNA with Maxima^TM^ H Minus cDNA Synthesis Master Mix with dsDNase (Thermo scientific) and stored at −80°C for fluorescence quantitative detection.

### Real Time Quantitative PCR

Based on gene sequence information in GenBank, specific primers for the IFN, ISG, and ARV σ*C* genes were designed (Table 1). All primers were synthesized by Invitrogen. ARV σ*C* genes were amplified and cloned into the pMD18-T vector (TaKaRa, Dalian, China), and the constructed recombinant plasmid was designated σ*C*-pMD18-T. The σ*C*-pMD18-T recombinant plasmid was used to generate the standard curve as described previously (31). The transcription levels of *IFN, ISG*, and ARV σ*C* genes were quantified by qPCR with PowerUp^TM^ SYBR^TM^ Green Master Mix (Thermo Scientific) in a QuantStudio 5 real-time PCR system (Thermo Lifetech ABI). *GAPDH*, a housekeeping gene, served as the internal control to normalize the relative expression levels of the detected genes. The real time quantitative PCR mix had 20 μL, including 10 μL of the SYBR mix, 1 μL of each of the upstream and downstream primers at the final concentration of 0.5 μM, and 2 μL of the cDNA template. The thermal cycling program was 50°C activation for 2 min, 95°C predenaturation for 5 min, and 40 cycles of 95°C denaturation for 15 s, annealing at 60°C for 1 min, and extension at 72°C for 10 s. After the reaction was completed, melting curve analysis was performed at 95°C for 15 s, 60°C for 1 min, and 95°C for 1 s.

**Table 1:** Primers used in this study.

**Gene**	**Genbank accession number**	**Primer sequences (5^**′**^-3^**′**^)**	**References**
ARV *σC*	L39002.1	F:CCACGGGAAATCTCACGGTCACT, R:TACGCACGGTCAAGGAACGAATGT	
*IFNA*	AB021154.1	F:ATGCCACCTTCTCTCACGAC, R: AGGCGCTGTAATCGTTGTCT	(25)
*IFNB*	X92479.1	F:ACCAGGATGCCAACTTCT, R:TCACTGGGTGTTGAGACG	(26)
*OAS*	NM_205041.1	F:GCGGTGAAGCAGACGGTGAA, R:CGATGATGGCGAGGATGTG	(27)
*GAPDH*	NM_204305.1	F:GCACTGTCAAGGCTGAGAACG, R:GATGATAACACGCTTAGCACCAC	(27)
*IFITM3*	KC876032.1	F:GGAGTCCCACCGTATGAAC, R:GGCGTCTCCACCGTCACCA	(28)
*MX*	AY695797.1	F:AACGCTGCTCAGGTCAGAAT, R:GTGAAGCACATCCAAAAGCA	(29)
*PKR*	AB125660.1	F:CCTCTGCTGGCCTTACTGTCA, R:AAGAGAGGCAGAAGGAATAATTTGCC	(30)
*VIPERIN*	EU427332.1	F:CAGTGGTGCCGAGATTATGC, R:CACAGGATTGAGTGCCTTGA	
*IFIT5*	XM_421662	F:CTCCCAAATCCCTCTCAACA, R:AAGCAAACGCACAATCATCA	
*ISG12*	BN000222.1	F:TCCTCAGCCATGAATCCGAACA, R:GGCAGCCGTGAAGCCCAT	
*ZFP313*	AY604724.1	F:ATCGCTTTACCTTTCCTTG, R:GTGCCATCGTATCATCTTCA	
*DDIT4*	XM_015288240.1	F:CGACCTCTGCGTGGAGCA, R:CAGGGACTGGCCGAAAGC	
*IFI6*	NM_001001296.5	F:CACTCCTCAGGCTTTACC, R:GACCGATGCTTCTTTCTATT	
*CD47*	AY234188.1	F:GCTTTCAAGTTGTGGGTT, R:TGCAGTAGGTTCGGTCTC	

### Statistical Analysis

The cycle threshold (CT) value of the ARV σ*C* gene was input into the standard curve to calculate the viral copy number as described previously (31), and analyze ARV replication in SPF chickens.

The CT values of the target genes (IFNs and ISGs) and the internal control gene (*GAPDH* gene) obtained from real time quantitative PCR were used to calculate relative expression levels of target genes at different time points using the 2^−ΔΔCT^ method (32). First, sample differences of internal reference gene homogenization, ΔCt = Ct value (target gene)–Ct value (GAPDH gene). Second, comparison of infected samples and control samples, ΔΔCt = ΔCt values (infected samples) – ΔCt values (control samples). Third, calculate the target gene changes in transcription levels, times change = 2^−ΔΔCT^. The target gene times change were used for statistical analysis. Statistical analysis was performed within IBM SPSS Statistics 2.0. Student's *t*-test was run to assess differences. *p* < 0.05 indicated that a difference between the experimental group and the control group was significant, in which case the corresponding figure was labeled with ^*^. *p* < 0.01 indicated that the difference between the experimental group and the control group was very significant (^**^ in the figures). Figures were plotted by GraphPad Prism 8 software.

## Results

### Pathological Changes in Joints After ARV Infection

**Figure 1:**
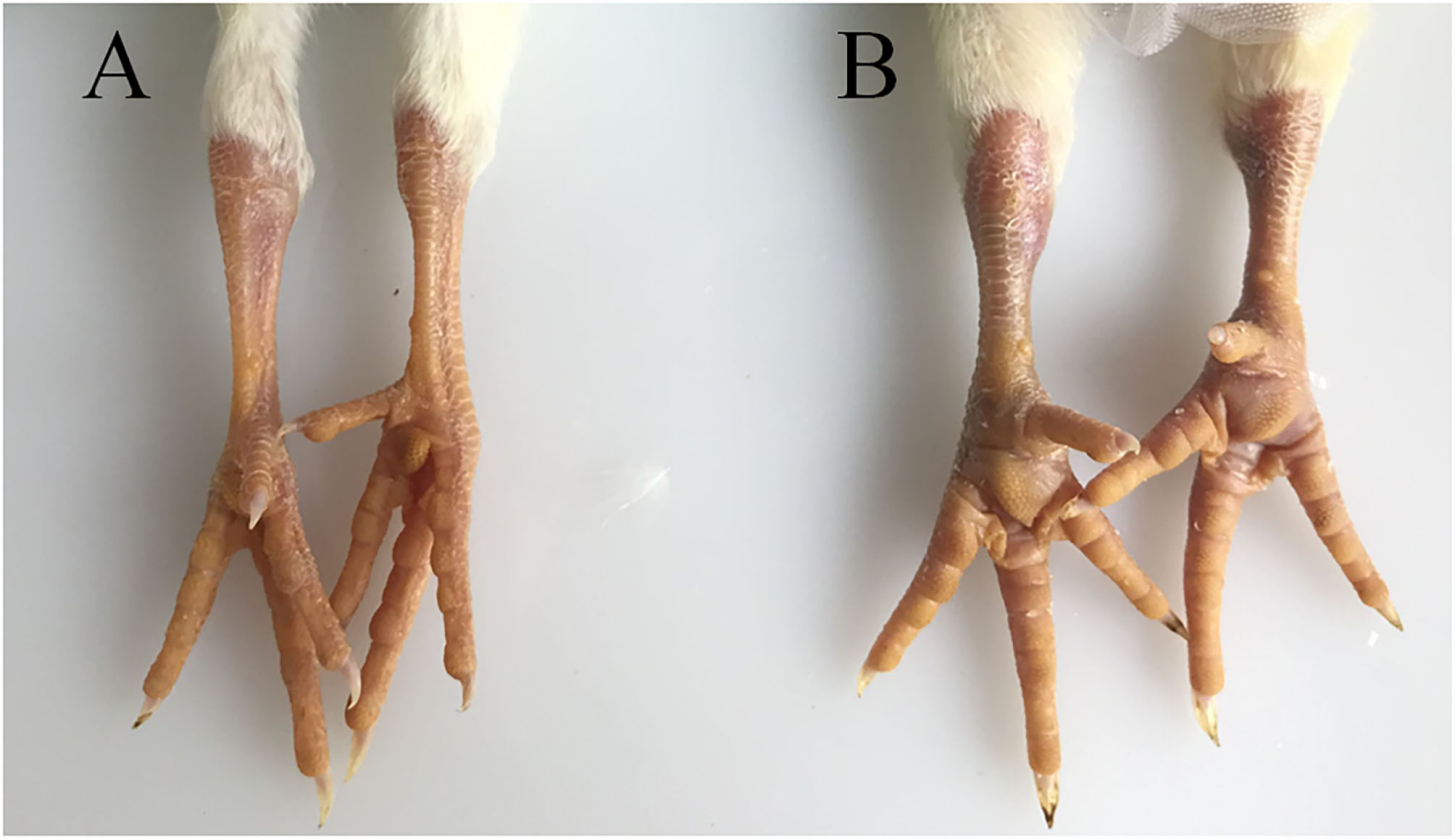
Pathological changes in SPF chicken joints after ARV infection. **(A)** Control group, chickens were injected with 0.1 ml PBS in the foot pad. **(B)** Experimental group, chickens were challenged with 0.1 ml of 10^4^ median tissue culture infectious doses/0.1 ml ARV S1133 through foot pad injection.

The hock joints are the major target organ of ARV. The hock joints of SPF chickens in the infection group showed redness and swelling (Figure 1). Dissection results showed that exudates in the articular cavity of the hock joints of chickens in the infection group increased. Throughout the experimental process, the foot pads and hock joints of SPF chickens in the control group were all normal and did not change significantly.

**Figure 2:**
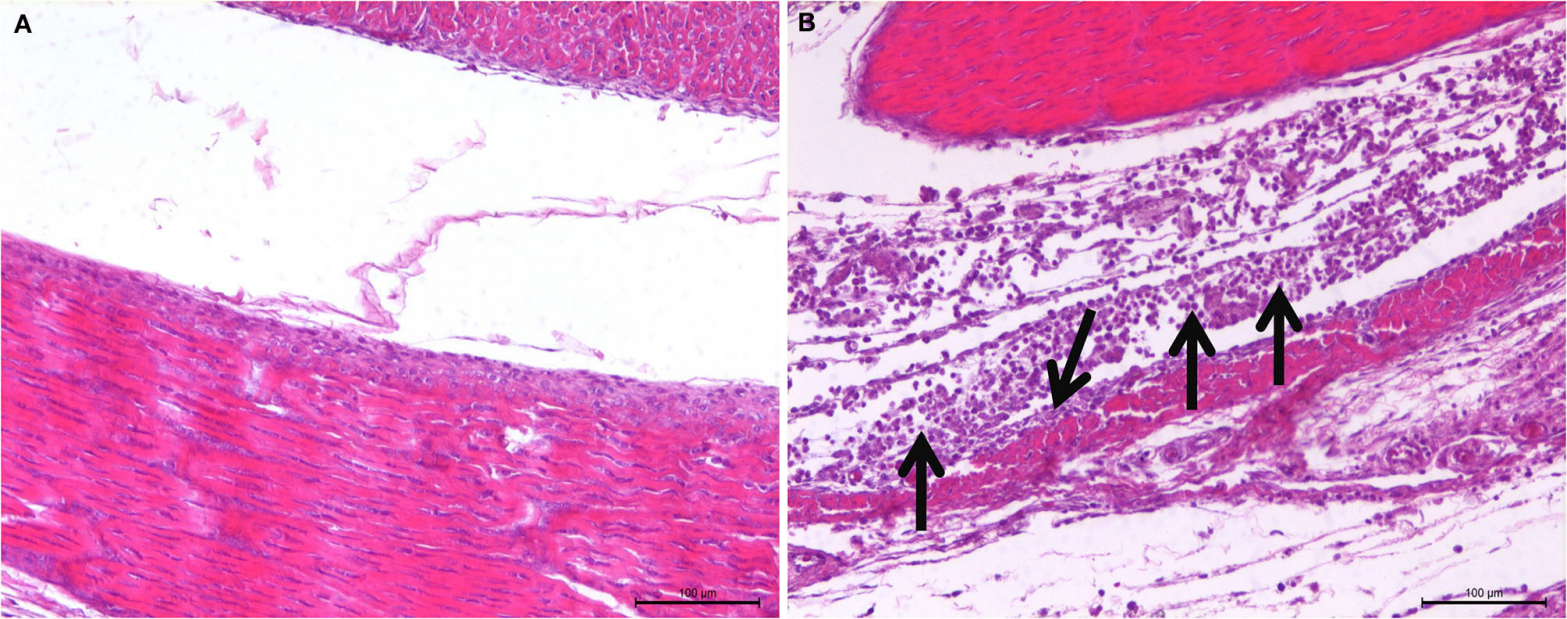
Histopathological changes of SPF chicken joints after ARV infection. **(A)** Control group, chickens were injected with 0.1 ml PBS in the foot pad. **(B)** Experimental group, chickens were challenged with 0.1 ml of 10^4^ median tissue culture infectious doses/0.1 ml ARV S1133 through foot pad injection. The arrow indicates tendon fibroblasts and synovial epithelial cells were degenerated and necrotic, and a large number of infiltrated inflammatory cells.

The observation results of the pathological histology sections are shown in Figure 2. In the joint, tendon fibroblasts and synovial epithelial cells were degenerated and necrotic, and a large number of infiltrated inflammatory cells, mainly monocytes and macrophages, were noted.

### Viral Loads in Joints After ARV Infection

**Figure 3:**
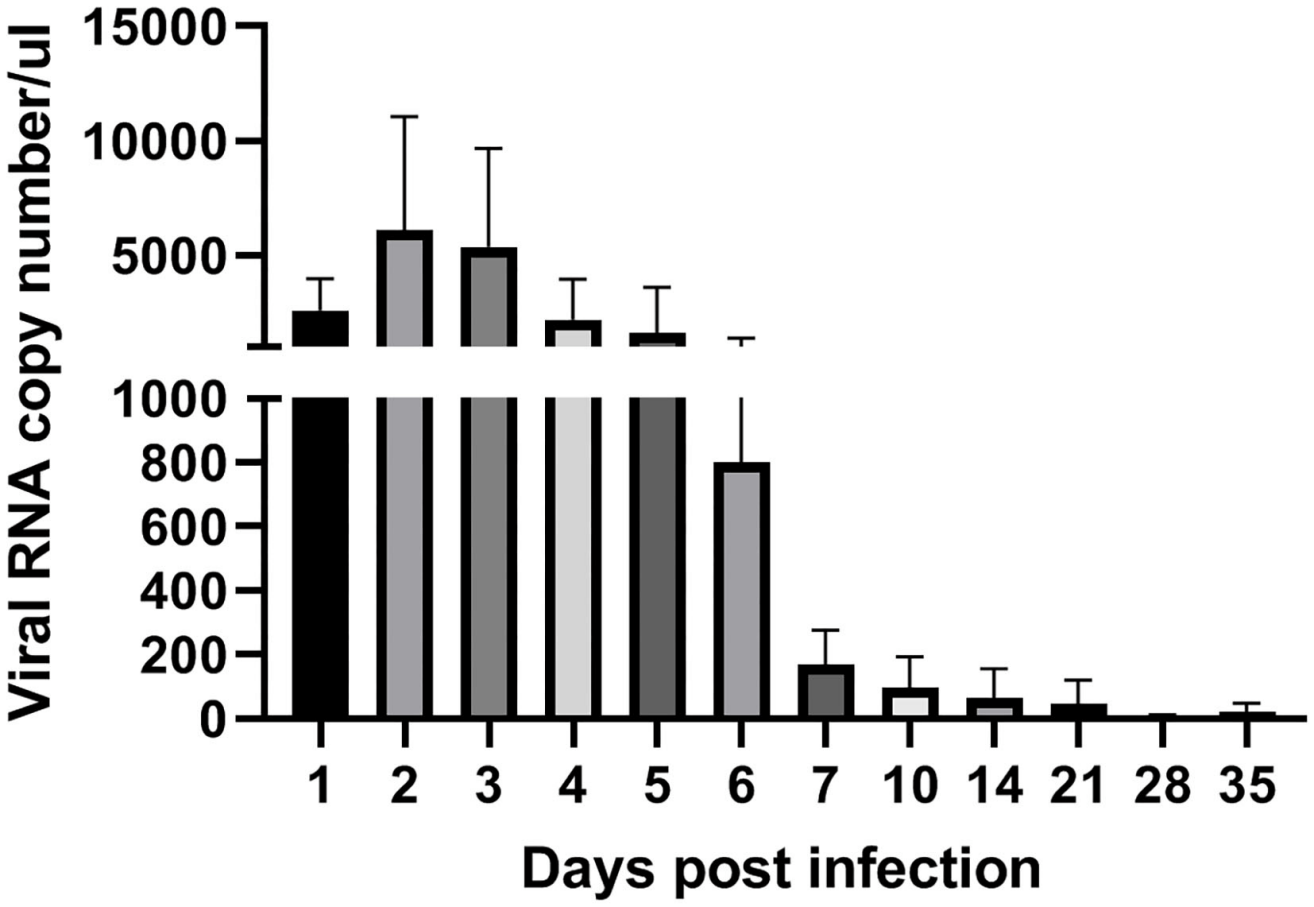
Kinetics of ARV replication in SPF chicken joints after infection. Chickens were challenged with 0.1 ml of 10^4^ median tissue culture infectious doses/0.1 ml ARV S1133 through foot pad injection. At 1, 2, 3, 4, 5, 6, 7, 10, 14, 21, 28, and 35 days postinfection (dpi), three chickens in each group were randomly collected for ARV viral loads detection by real time quantitative PCR detection. Sampling was 30 mg joint (tendon, synovium, and 133 articular cartilage). The cycle threshold (CT) value of the ARV σ*C* gene was input into the standard curve to calculate the viral copy number. Viral copy number expressed as ARV copies in 1 μL genome equivalent (150 ngRNA, about 1 mg joint tissues). Figures were plotted by GraphPad Prism 8 software.

To analyze ARV proliferation in joints, the expression level of the ARV σ*C* gene was detected by real time quantitative PCR to measure the viral load of ARV. The detection results (Figure 3) showed that ARV rapidly proliferated starting at 1 dpi. The viral load peaked by three dpi and was still high six dpi. The ARV viral load rapidly decreased starting on day 7. The control group was negative for the ARV σ*C* gene throughout the experimental process.

### Changes in Transcription Levels of IFNs in Joints After ARV Infection

**Figure 4:**
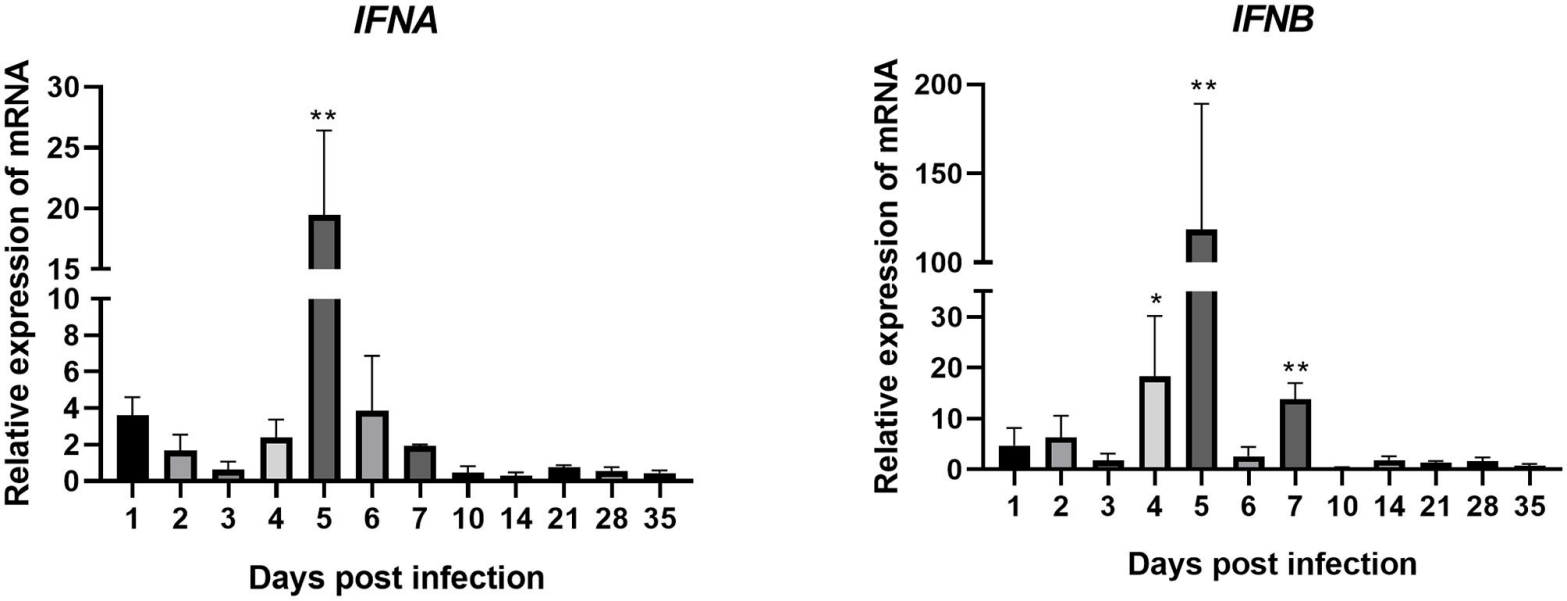
Changes in the transcription levels of IFNs in joints after ARV infection. Chickens were challenged with 0.1 ml of 10^4^ median tissue culture infectious doses/0.1 ml ARV S1133 through foot pad injection. At 1, 2, 3, 4, 5, 6, 7, 10, 14, 21, 28, and 35 days postinfection (dpi), three chickens in each group were randomly collected for IFNs detection by real time quantitative PCR detection. IFN gene changes in transcription levels =2–^ΔΔCT^. Relative gene expression was expressed as fold of control group samples. Statistical analysis was performed within IBM SPSS Statistics 2.0. Student's *t*-test was run to assess differences. Figures were plotted by GraphPad Prism 8 software. Asterisks indicate significant differences (*P < 0.05, **P < 0.01).

*IFNA* expression in joints of the experimental group was upregulated between 1 and 7 dpi (except for 3 dpi, when it was downregulated) after artificial infection with ARV (Figure 4). The expression level peaked on 5 dpi at 20 times that in the control group (*P* < 0.01). After 7 dpi, its expression went down.

The expression of *IFNB* was very similar to that of *IFNA*. It was upregulated between 1 and 7 dpi, peaking at 5 dpi at 118 times that in the control group (*P* < 0.01). The expression levels of *IFNB* at 4 and 7 dpi were also very high, at 18 and 14 times those in the control group, respectively (both *P* < 0.01). These genes were all downregulated after 7 dpi. These results indicate that, after ARV infection. The expression levels of *IFNB* and *IFNB* (type I IFNs) were both upregulated, especially *IFNB*.

### Changes in Transcription Levels of ISGs in Joints After ARV Infection

**Figure 5:**
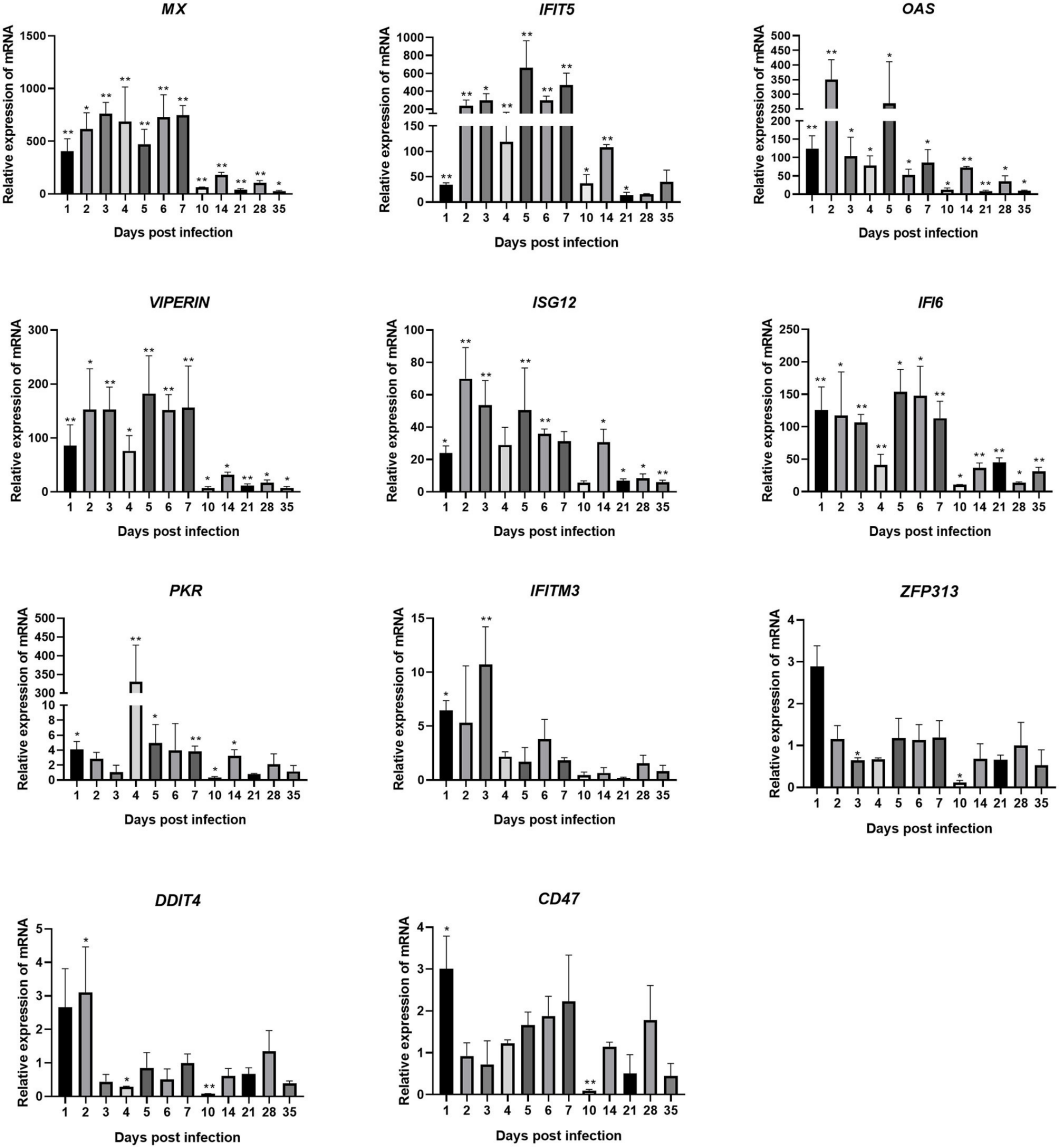
Changes in the transcription levels of ISGs in joints after ARV infection. Chickens were challenged with 0.1 ml of 10^4^ median tissue culture infectious doses/0.1 ml ARV S1133 through foot pad injection. At 1, 2, 3, 4, 5, 6, 7, 10, 14, 21, 28, and 35 days postinfection (dpi), three chickens in each group were randomly collected for ISGs detection by real time quantitative PCR detection. ISGs gene changes in transcription levels =2–^ΔΔCT^. Relative gene expression was expressed as fold of control group samples. Statistical analysis was performed within IBM SPSS Statistics 2.0. Student's *t*-test was run to assess differences. Figures were plotted by GraphPad Prism 8 software. Asterisks indicate significant differences (*P < 0.05, **P < 0.01).

Changes in the transcription levels of ISGs induced by IFNs are shown in Figure 5. The results showed that ARV infection rapidly and significantly upregulated the mRNAs of 11 common avian ISGs in joints. The expression levels of ISGs *MX, IFITM3, IFIT5, OAS, PKR, VIPERIN, ISG12, IFI6*, and *CD47* were upregulated on days 1–7 of infection. *ZFP313* and *DDIT4* expression suddenly increased on day 1 or 2 and then decreased. The expression levels of increase of *MX, IFIT5, OAS, VIPERIN, ISG12*, and *IFI6* were the most significant (several hundred-fold), and their expression levels throughout the experimental process were all higher than those in the control group (*P* < 0.01). *MX* expression stayed at a higher level between 1 and 28 dpi. Its expression on 1 dpi was 403 times that in the control group, and it was even higher afterward. Its expression level on 7 dpi was the highest, at 748 times that in the control group, and it began to decrease afterward. The expression levels of *IFIT5* and *ISG12* were both rapidly upregulated on 1 dpi. *IFIT5* expression peaked at 5 dpi at 661 times the control level. *ISG12* expression peaked at 2 dpi at 70 times the control level. The expression of *IFIT5* and *ISG12* were both upregulated between 2 and 7 dpi. *OAS* expression was also significantly upregulated at 1 dpi at 125 times higher than the control level. It peaked at 2 dpi at 350 times the control level. Except at 10 and 21 dpi, *PKR* expression was upregulated at all time points. Overall, its levels of upregulation were not very high, though interestingly, at 4 dpi it was at 331 times the control level. The expression levels of *VIPERIN* and *IFI6* were upregulated at 1 dpi and stayed upregulated between 1 and 7 dpi. The expression levels of *IFITM3, ZFP313, DDIT4*, and *CD47* were rapidly upregulated in the early stage of infection, though not very highly. They were downregulated after 1–3 days of infection to levels similar to those in the control group (except that the expression levels of *ZFP313, DDIT4*, and *CD47* had significant downregulation at 10 dpi).

## Discussion

ARV was first isolated in 1954 (33). Current studies mainly focus on its etiology and epidemiology, and there have been relatively few studies about its pathogenesis and regulatory signaling pathways that induce innate immunity. After invasion of a host, a virus is recognized by pathogen-associated pattern molecules to induce the body to produce type I IFNs to further induce ISG production, thus eventually exerting the antiviral effect (34). Therefore, analysis of changes in the transcriptional expression levels of ISGs after ARV infection is important to better understand the pathogenesis of ARV, and may be helpful for determining the ISGs that may be involved in the anti-viral defense against ARV. IFN-α and IFN-β are type I IFNs that play important roles in the antiviral process. The main symptom of ARV infection is viral arthritis. Therefore, joints are the major target organ of this virus. Our real time quantitative PCR showed that both of these type I IFNs expression were upregulated in joints of SPF chickens after ARV S1133 infection, indicating that ARV infection can upregulate *IFN* expression. These results are consistent with previous results (changes in IFNs after ARV infection in CEF cells) (21). This upregulated expression occurred in the early stage of viral infection. The expression level of *IFNB* (118.5-fold on 5 dpi) was significantly higher than that of *IFNA* (19.5-fold on 5 dpi). Therefore, we speculate that the body mainly exerts antiviral functions through IFN-β after ARV infection.

This study showed that mRNA expression levels of many ISGs in joints showed a pattern of changes after SPF chickens were infected with ARV S1133. The trend of changes was basically consistent with the viral load in joints (when the copy number of ARV increased, the mRNA transcription levels of the ISGs increased correspondingly; when ARV propagation was inhibited, the mRNA transcription levels of the ISGs decreased accordingly) and was associated with dynamic pathological changes in the joints. In SPF chicken joints, the transcription levels of *IFNA, IFNB*, and ISG genes, including *MX, IFITM3, PKR, OAS, IFIT5, ISG12, VIPERIN, IFI6*, and *CD47*, were upregulated between 1 and 7 dpi. The ARV viral load in chicken joints rapidly increased after 1 day of viral challenge. The viral load was high within the first 6 days of viral challenge but rapidly decreased starting on day 7. These results suggest that days 1–7 after ARV infection are the critical period of anti-ARV immune responses in the body. *IFNA, IFNB*, and ISGs, including *MX, IFITM3, PKR, OAS, IFIT5, ISG12, VIPERIN, IFI6*, and *CD47*, play important roles in defending against ARV invasion, inhibiting ARV replication and propagation, and viral clearance. Among the expression upregulated ISGs, *MX, IFIT5, OAS, VIPERIN, ISG12*, and *IFI6* had the largest expression levels of upregulation (hundreds-fold), and these ISGs all showed a certain pattern of changes: persistent upregulation in the early stage of infection, peaking 3–5 days after infection. Accordingly, with expression increases in ISGs, the viral load of ARV began to decrease. These results indicate that these ISGs are important in the anti-ARV infection process in hosts, and these ISGs (*MX, IFIT5, OAS, VIPERIN, ISG12*, and *IFI6*) will be screened by overxpression and silencing experiments to determine the specific anti-ARV ISG in our further study.

The results of this study are similar to those of our previous study (27) considering the innate systemic immune responses. Our previous study (27) showed that expression levels of type I IFNs, including *IFNA* and *IFNB*, type II IFN IFN-γ, and the ISGs *MX, IFITM1*, and *OSAL* were all upregulated in peripheral-blood lymphocytes of SPF chickens in the early stage of ARV infection and peaked 3 days after infection. IFN-β induces significant upregulation of MX, OAS, and PKR expression (13, 18, 35) to defend against infection by various viruses. The expression levels of *MX, OAS*, and *PKR* were significantly upregulated in this study as well. These experimental results enrich our understanding of the innate immune response mechanisms of hosts in defending against ARV infection and provide a theoretical basis for prevention and control of ARV infection.

All ISG do not exhibit the same mechanism of anti-viral activity. Indeed the ISGs that might be involved in anti-viral response differ according to the virus. Shah et al. (36) confirmed that only the ISG protein VIPERIN was the anti-Newcastle disease virus ISG. In this study, the anti-ARV activity and the ARV replication inhibition activity of the ISGs that were significantly upregulated or downregulated and the mechanisms by which ARV antagonizes the antiviral effects of these ISGs still require further research.

An interesting phenomenon in this study was that the levels of changes in the expression of dsRNA-dependent protein kinase (*PKR*) did not change much throughout the experiment except that at 4 dpi it suddenly increased to 331 times the control level. This expression level of upregulation was far higher than those of other ISGs on 4 dpi. PKR is an interferon-induced and dsRNA-activated serine/threonine kinase. ARV σA, infectious bursa disease virus VP3, and influenza virus NS1 can all interact with dsRNA to inhibit PKR activation and hinder the antiviral function of IFNs (19, 20, 37). The meaning of the sudden, high *PKR* expression upregulation in joints at 4 dpi after ARV infection requires further investigation.

## Data Availability Statement

The original contributions presented in the study are included in the article/Supplementary Material, further inquiries can be directed to the corresponding author/s.

## Ethics Statement

The animal study was reviewed and approved by Animal Ethics Committee of the Guangxi Veterinary Research Institute.

## Author Contributions

ZX and LZ designed and coordinated the study and helped to review the manuscript. SW performed the experiments, analyzed the data, and wrote the manuscript. LX analyzed the data and wrote the manuscript. LW, JH, XD, ZqX, SL, TZ, YZ, and MZ participated in the Animal experiments. All authors contributed to the article and approved the submitted version.

## Conflict of Interest

The authors declare that the research was conducted in the absence of any commercial or financial relationships that could be construed as a potential conflict of interest.
